# Unveiling ncRNA regulatory axes in atherosclerosis progression

**DOI:** 10.1186/s40169-020-0256-3

**Published:** 2020-02-03

**Authors:** Estanislao Navarro, Adrian Mallén, Josep M. Cruzado, Joan Torras, Miguel Hueso

**Affiliations:** 1 Independent Researcher, Barcelona, Spain; 2 Department of Nephrology, Hospital Universitari Bellvitge and Bellvitge Research Institute (IDIBELL), C/Feixa Llarga, s/n; L’Hospitalet de Llobregat, 08907 Barcelona, Spain

**Keywords:** Dark transcriptome, miRNA, lncRNAs, Alternative 3′UTRs, Regulatory RNA networks, Atherosclerosis

## Abstract

Completion of the human genome sequencing project highlighted the richness of the cellular RNA world, and opened the door to the discovery of a plethora of short and long non-coding RNAs (the dark transcriptome) with regulatory or structural potential, which shifted the balance of pathological gene alterations from coding to non-coding RNAs. Thus, disease risk assessment currently has to also evaluate the expression of new RNAs such as small micro RNAs (miRNAs), long non-coding RNAs (lncRNAs), circular RNAs (circRNAs), competing endogenous RNAs (ceRNAs), retrogressed elements, 3′UTRs of mRNAs, etc. We are interested in the pathogenic mechanisms of atherosclerosis (ATH) progression in patients suffering Chronic Kidney Disease, and in this review, we will focus in the role of the dark transcriptome (non-coding RNAs) in ATH progression. We will focus in miRNAs and in the formation of regulatory axes or networks with their mRNA targets and with the lncRNAs that function as miRNA sponges or competitive inhibitors of miRNA activity. In this sense, we will pay special attention to retrogressed genomic elements, such as processed pseudogenes and Alu repeated elements, that have been recently seen to also function as miRNA sponges, as well as to the use or miRNA derivatives in gene silencing, anti-ATH therapies. Along the review, we will discuss technical developments associated to research in lncRNAs, from sequencing technologies to databases, repositories and algorithms to predict miRNA targets, as well as new approaches to miRNA function, such as integrative or enrichment analysis and their potential to unveil RNA regulatory networks.

## Background. Atherosclerosis progression and the dark transcriptome

Atherosclerosis (ATH) is a complex inflammatory disease of the vessel wall caused by a combination of multiple factors including genomics, epigenetic modifications and environmental conditions, that place an enormous burden on modern societies, particularly in the aging population [1]. The complexity of its causes and mechanisms makes ATH prevention and treatment largely ineffective, becoming an enormous challenge for our society, favored by our lifestyle [2, 3]. Thus, there is an urgent need to develop a more personalized medicine, and to enhance patient care through improved diagnostic sensitivity with more effective interventions in ATH prevention and treatment [4]. In this sense, years of research on the genomic basis of ATH have provided the biomedical community with a knowledge of gene-related ATH risk factors, such as SNPs [5, 6], genes and gene variants [7–9], alterations in DNA methylation [10, 11], changes in gene expression [12, 13], etc. Nevertheless, in the last years a new player has entered the game of disease-associated genes: the highly heterogeneous group of non-coding RNAs, which are progressively becoming important factors for atherosclerosis (and other diseases) research either as biomarkers of disease progression or as pathophysiological intermediates, while their operative interactions highlight the remarkable structural and functional complexity of the human genome.

### From junk to gold, non-coding RNAs are functional components of the human transcriptome

Analysis of the sequenced human genome showed that over 80% of the genome could be considered as biochemically active [14], most of it in the form of DNase I-accessible loci or candidate regulatory sequences [15–17]. Although the number of protein coding genes in the human genome has been recently estimated at 20–25,000 [18, 19], the total number of active genomic loci is significantly higher, with a best guess being close to 10e5 [20] most of them corresponding to a plethora of heterogeneous, non-protein-coding, RNAs [21]. Originally considered as part of the “dark transcriptome” or “genomic dark matter”, i.e. genomic sequences of uncertain or unknown function [22, 23], non-coding RNAs were initially classified by their length into short (< 200 nucleotides long) and long (lncRNAs, > 200 nucleotides long) RNAs. Although some efforts have been devised to make a more informative and standardized nomenclature of ncRNAs [24, 25], this primary classification based in length is still widely accepted by the scientific community, and we will follow this convention in this review. Short ncRNAs include the already known snRNAs, snoRNAs and tRNAs, the PIWI-associated RNAs that repress expression of transposable and repetitive elements in the germline to maintain genomic stability [26] and the microRNA (miRNA) family of translational regulators (see the “MicroRNAS (miRNAs), a family of pleiotropic translational regulators” section here and [27] for a review). On the other hand, lncRNAs conformed a highly heterogeneous group in size and function, with regulatory roles in development, differentiation and disease progression [28–31], and whose expression is frequently altered in disease (see “Long non-coding RNAs (lncRNAs) and their functional relationship with miRNAs” section here and [32] for a review).

**Fig. 1:**
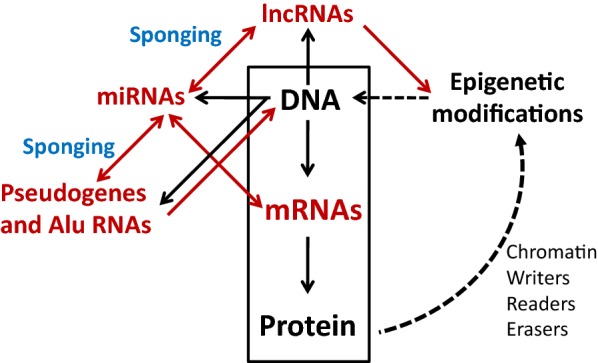
The “central dogma” revisited. Shown are new additions to the central dogma (boxed), highlighting the fundamental role of miRNAs in the regulation of gene expression. This work reviews the functional relationships among the RNAs drawn in red. Black arrows means transcription or translation, double-headed red arrows mean mutual interactions, red arrows functional interactions (in the case of pseudogenes and Alu elements, retroinsertion) and dotted lines refer to the histone code and chromatin modifications

Data on the expression of non-coding RNAs have drawn a new model of the human genome function in which the nucleus is pervasively transcribed, even in intronic and intergenic sites [33], to generate a complex population of short and long non-coding RNAs with putative regulatory functions [34]. Although this model has been challenged on technical bases [35, 36], it is now widely accepted that in the mammalian genome over one order of magnitude more genomic sequence is transcribed to non-coding RNA than to protein-coding RNA [37]. This new model has also changed the original paradigm on the flow of genetic information from the linear “DNA makes RNA makes protein”, for many years considered as the central dogma of molecular biology [38, 39], to a multilayered process characterized by the pervasive expression of many structural or regulatory RNAs with the ability to establish different tiers of functional interactions (Fig. 1). This change of paradigm has had a number of consequences, such as the exponential increase in the number of non-protein coding RNAs associated to diseases, drawing new layers of epigenetic control that confer regulatory plasticity and are deregulated in disease, and the need to profile and give sense to these expression alterations and to the huge amount of expression data generated by disease-associated sequencing projects.

Our group is interested in the role of ncRNAs in the context of ATH progression. Here, we will review recent developments on the impact of non-coding RNAs on ATH progression, focusing on the role of microRNAs. We will also study their functional relationship with lncRNAs, since these have been reported to play key functions in physiology and disease [40], and to have a role in miRNA function as miRNA “sponges” or competitive inhibitors of miRNA activity. Furthermore, in this group of lncRNAs we will also include other transcripts, such as pseudogenes, and expressed Alu elements, which have been reported to also interact with miRNAs but that have been less studied.

## DNA sequencing and the integration of transcriptomics with personalized medicine

In less than 25 years, DNA sequencing [41, 42] evolved from a technique only available to the elite of basic research laboratories to a tool widely used in clinical settings, a technical evolution that crystallized in the sequencing of the human genome by two scientific consortia [43, 44] and opened the age of the personalized genomic medicine. Today, systematic DNA sequencing of whole genomes or exomes is performed in all branches of medicine as a prognostic or diagnostic tool, or to follow treatment or disease progression. Furthermore, single-cell RNA-sequencing (scRNA-seq) methodologies allow the genome-wide profiling of individual cells to identify mutations and to characterize and quantify cellular heterogeneity and its variations in disease [45].

### Open sequence repositories, the key to the sequencing revolution

One key factor of success of the sequencing revolution has been the almost immediate accessibility to all sequences generated in research laboratories, many times even prior to publication. This was possible because of the establishment of three mirrored sequence repositories (GenBank at the NCBI, DNA DataBank of Japan and the European Nucleotide Archive, Table 1) that stored, annotated and provided public and unrestricted access to all DNA and RNA sequences in the context of the International Nucleotide Sequence Database Collaboration [46]. One critical point of these repositories is that these not only facilitated the diffusion of DNA/RNA sequences by giving each one of them a unique sequence identifier, but also created a database of “reference genomes”, a collection of non-redundant, reference genomic, transcriptomic and protein sequences, intended to function as primary sequence references in genomic works [47, 48]. Furthermore, these also provided diverse annotations to the sequences, from functional domains to genomic loci, intended as maps of the genomic landscape to facilitate the interpretation of the genomic context of a specific sequence [20]. Lastly, all these information have been integrated in “genomic browsers” (Ensembl [49], and NCBI’s genome viewer [50]) which allow users going from chromosome regions to the sequence of any transcriptional unit and its variants.

**Table 1:** Sequence databases and repositories

Sequence repository or database	Web page address
Genbank	-www.ncbi.nlm.nih.gov
DDBJ, DNA Databank of Japan	-www.ddbj.nig.ac.jp
ENA, European Nucleotide Archive	-www.ebi.ac.uk/ena
INSDC (and Sequence Read Archives)	-insdc.org
NCBI Reference Sequence Database (Refseq)	-www.ncbi.nlm.nih.gov/refseq/
Ensembl Genome Viewer	-www.ensembl.org
Genome Data Viewer	-www.ncbi.nlm.nih.gov/genome/gdv/
miRBase	-www.mirbase.org
LNCipedia Project (database of human ncRNAs)	-www.lncipedia.org
NONCODE (knowledge database of ncRNAs)	-www.noncode.org
RNA central (ncRNA sequence database)	-rnacentral.org
ENCODE (encyclopedia of DNA elements)	-www.encodeproject.org
FANTOM (functional annotation of the human genome	-fantom.gsc.riken.jp

Shown are repositories, databases data viewers of nucleic acids. This is not an exhaustive list, and the selection only reflects authors’ preferences

### The revolution in sequencing technologies

Initial sequencing protocols used ultrathin PAGE gels to resolve radioactively-labelled fragments [51, 52]. Although DNA sequencing was subsequently improved by the introduction of fluorescent labels [53] and by the use of the thermostable Taq DNA polymerase [54, 55], these methods were not adaptable to the high throughput-approach requisites of clinical sequencing. In this context, sequencing of the human genome started a race for new methods and faster and cheaper sequencing machines, with the objective set at the “1000 $ genome” [56], that prompted different approaches to the high-throughput sequencing of DNA. Aside of the pore sequencing (Oxford Nanopore) that perform a direct sequencing by using protein nanopores without DNA synthesis or amplification [57], most of the current sequencing platforms use a highly/mass parallel approach [58]. In this approach, the original sample (genomic DNA for genome sequencing or RNA copied as cDNA for exome sequencing) is fragmented and the fragments immobilized in individual cells where they are amplified, cycle-copied with labeled nucleotides and each reaction is individually detected as fluorescence (Illumina, Qiagen Gene reader or Pacbio platforms), or as H^+^ (Ion torrent platform). Lastly, each sequence is compared with reference genomes or exomes for identification [58].

### The technical challenge of sequencing ncRNAs

Sequencing ncRNAs suppose a technical challenge derived of their heterogeneous length and exonic composition since these have sizes ranging from the 22 nucleotides of mature miRNAs [59] to the 22.7 kb of the single exon NEAT1_v2 transcript [60]. One possibility to overcome this problem is performing short sequence reads, like the expressed sequence tags (ESTs) in which individual cDNA clones were sequenced by their 3′ end only, generating reads of a few hundred nucleotides that were as “tags” of the full-length transcript [61]. Although this approach is suitable for the construction of genetic and physical maps of expressed sequences [62–64], it would not detect all the richness of CDS mutations (required for cancer research) or the complex patterns of alternative splicing that display lncRNAs. In this sense, and as an example, the relatively “short” 3.8 kb ANRIL [65], is expressed as over 50 splicing isoforms, linear or circular [66, 67], some of them disease-related [68]. In this complex context it is evident that recovering most of the lncRNA genomic information will require not only developing new sequencing hardware able to provide longer and more accurate reads, but also to improve the ability of reverse transcriptase (RT) to copy as much as possible of the full-length sequence, although these problems could be circumvented by a more “classical” molecular biology approach using random primers for the RT reaction, followed by the painstaking growth of the sequence by the 5′/3′ RACE (rapid amplification of cDNA ends) technique [69].

On the other hand, and for the case of small miRNAs, the entire population of a tissue can be sequenced by gel-purifying the fraction of small RNAs, adding them 5′ and 3′ adaptors with T4 RNA ligase, followed by a reverse transcription and PCR amplification prior to sequencing in any of the above platforms. In this way, representative results are obtained even for low expressed miRNAs, since the number of reads is proportional to the number of initial miRNA copies [70]. Furthermore, when dealing with miRNAs, the new high-throughput sequencing techniques have the potential to provide single-nucleotide resolution of miRNA species, facilitate de novo miRNA discovery and offer a dynamic range for miRNA quantification [71].

## MicroRNAS (miRNAs), a family of pleiotropic translational regulators

MiRNAs are small RNAs (over 22 nucleotides long) with important roles in post-transcriptional gene regulation [59]. MiRNA genes are under transcriptional control, are transcribed by RNA polymerase II and suffer a process of maturation from pri-miRNA primary transcripts to the fully functional mature miRNAs which include activity of RNase III endoribonucleases DROSHA and DICER (see [72, 73] for reviews). In a recent work, Alles et al. estimated the entire human miRNAome as being composed by 2300 mature miRNAs of which 1115 were annotated in the version 22 of the specific miRNA database, miRbase [74]. MiRNAs function by targeting mRNAs, usually by base-pairing at their 3′UTR, for degradation or translational repression through the RISC complex (RNA Induced Silencing Complex) [27]. Recent reports estimated that over 60% of mRNAs harbour miRNA binding sites at their 3′UTRs, highlighting the importance of this interaction for the fine-tuning regulation of translation [75, 76]. An interesting characteristic of miRNA function is their functional promiscuity. Since only 6 bases of miRNA/mRNA complementarity are enough for duplex formation [77], a single miRNA can target dozens of different mRNAs which in turn can be regulated by many different miRNAs, thus creating a complex regulatory network [78].

### Dynamics of 3′UTRs: more than a counterpart for miRNA function

**Fig. 2:**
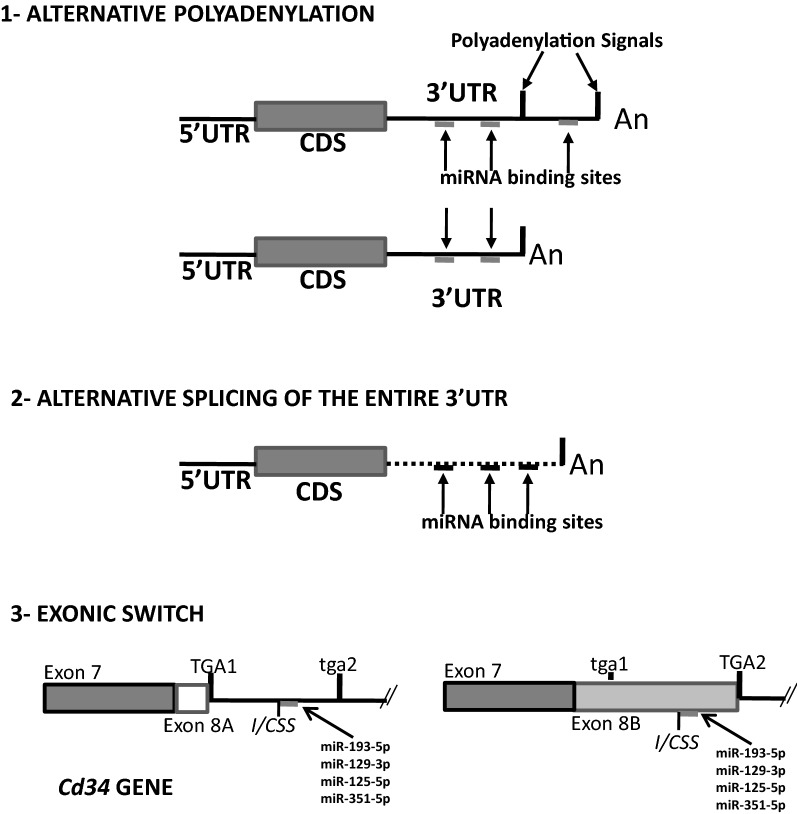
Impact of alternatively expressed 3′UTRs on their interaction with miRNAs. Shown are changes in the structure of the 3′UTRs with the potential to impact on the binding of specific miRNAs. **1**. The existence of alternative polyadenylation signals originate 3′UTRs of different lengths and different potential for miRNA binding. **2**. Alternative exons encoding different 3′UTRs differ in their potential for miRNA binding. **3**. Exonic switch. In the case of the Cd34 gene, an internal cryptic splice site (CSS) activates two different stop codons and generates two different exons 8, with the consequence that in one Cd34 isoform the binding site for a number of miRNAs is located in the 3′UTR, while in the other isoform it is located inside the CDS (taken from [87])

3′UTR regions of mRNAs are highly polymorphic in length and sequence, variations that may underlie changes in miRNA targeting and stability of the involved mRNAs [79]. Length polymorphisms of 3′UTRs are due to two different mechanisms: alternative splicing of untranslated exons, which is shared with most RNAs, and alternative polyadenylation, which seems to be mostly restricted to mRNAs, lincRNAs and NATs [80]. In a seminal work, Liaw et al. showed that cancer cells expressed shorter 3′UTRs than normal cells [81], suggesting that 3′UTR lengthening could constitute a mechanism to control accessibility to miRNA sites whose de-regulation could result in disease [82, 83], and suggesting that the 3′UTRome should be studied not only as a catalogue of miRNA binding sites but as a dynamic structure whose de-regulated changes could lead to the identification of new risk factors, or new candidates for disease genes [83]. Nevertheless, the effects of 3′UTR heterogeneity on the patterns of miRNA binding is a poorly studied topic, despite its potential importance, and there are only a few reports published. Without the aim of being exhaustive, since this topic will be treated more in deep in another work (Navarro et al. in progress), there are published examples on the regulation of miRNA activity by alternative 3′UTRs. In this sense, Xiao et al., showed that alternative polyadenylation at the 3′UTR of AAMDC originated two isoforms that differed in length and that only the long isoform was susceptible to miR-2428/664a silencing [84], while Bruhn et al. identified five different 3′-UTR length variants in the ABCB1 gene, of which only the three longer fragments harbored miRNA binding sites [85], and Pereira et al. working on the transcription factor Nurr1 (NR4A2), from the superfamily of nuclear receptors identified a number of 3′UTR length variants in the rat Nurr1 mRNA and described the selective interaction of miR-93, miR-204 and miR-302d with the longest Nurr1 mRNA [86]. Lastly, we have recently shown that a splicing event at an internal/cryptic splice site of the murine Cd34 gene would regulate the differential accession of miRNA-125/351 to the 3′UTR or the CDS of the Cd34mRNA [87] (Fig. 2).

### MicroRNAs in ATH progression

There is already a corpus of literature on the genetics and epigenetics of ATH evolution (see [40, 88, 89] for recent reviews), so that here in this section and in the next sections we will review recent developments on the relationship among miRNAs and ATH onset and progression and will highlight their use as therapeutic tools. In this sense, there are sound evidences demonstrating the involvement of miRNAs in many of the pathological processes that occur in ATH, and hundreds of miRNAs have been reported as key regulators of lipid handling, inflammation and cellular behaviors such as proliferation, migration and phenotypic switch [90], with alterations in the expression of miRNAs being detected not only in primary tissues but also in serum [91], urine [92], and exosomes [93]. Many reports have been published assessing modulation of miRNA expression in human patients and in mice models of ATH, some of them described in relatively mechanistic depth [94]. Table 2 reports recent descriptions of ATH-associated miRNAs either in animal models or in samples from human patients, their mRNA targets validated by luciferase reporter assays (not from bioinformatics predictions) and the effects of their expression alterations on ATH progression. This highlights the complexity of the miRNA/mRNA system, with different miRNAs targeting the same mRNA (e.g. miR-103 and miR-647 vs. PTEN), and a single miRNA targeting different mRNAs with different phenotypic outputs (miR-370 vs. FOXO1 and TLR4).

**Table 2:** ATH-associated miRNAs, mRNA targets and the effects of their expression on ATH progression

miRNA	Target mRNA/s^a^	Effect of miRNAs on ATH progression	References
miR-9	Oxidized low-density lipoprotein (lectin-like) receptor 1 (OLR1)	ApoE-null mice (U/R protective)	[95]
miR-23a-5p	ATP-binding cassette transporter A1/G1 ABCA1/G1	U/R promotes macrophage-derived foam cell formation	[96]
miR-23b	Forkhead Box O4 (FoxO4)	U/R inhibited VSMC proliferation and migration	[97]
miR-25-3p	A disintegrin and metalloprotease 10 (Adam10)	ApoE-null mice (U/R protective)	[98]
miR-30-3p	Transcription factor 21 (TCF21)	U/R increases viability of HUVEC cells	[99]
miR-34a	BCL2 apoptosis regulator (BCL2)	D/R facilitated growth and blocked apoptosis in HAECs	[100]
miR-98	Receptor for ox-LDL 1 (LOX-1)	D/R inhibited foam cell formation and lipid accumulation in aortas of ApoE-null mice	[101]
miR-99a-5p	Homeobox A1 (HOXA1)	U/R inhibits proliferation and invasion of ASMCs	[102]
miR-103	Phosphatase and tensin homolog (PTEN)	D/R suppressed inflammation and ERS in ECs from ApoE-null mice	[103]
miR-124	MCL-1 apoptosis regulator (MCL-1)	U/R represses viability, migration and capillary structure formation in HMEC-1 cells. Sponged by lncRNA HULC	[104]
miR-135b	Erythropoietin receptor (EPOR)	C57BL/6J male mice (D/R protective)	[105]
miR-142-3p	Rapamycin-insensitive companion of MTOR (Rictor)	D/R inhibited ECs apoptosis and ATH development in HAECs	[106]
miR-223	Insulin growth factor-1 receptor (IGF-1R)	U/R inhibits foam cell formation in VSMCs of human ATH patients	[107]
miR-338-3p	BMP and activin membrane-bound inhibitor (BAMBI)	D/R promoted viability and inhibited apoptosis in ox-LDL-induced HUVECs	[108]
miR-365b-3p	A disintegrin and metalloproteinase with thrombospondin motifs 1 (ADAMTS1)	U/R attenuated PDGF-BB-induced proliferation and migration of HCASMCs	[109]
miR-370	Forkhead Box 1 (FOXO1)	U/R promotes invasion and proliferation of HUVECs	[110]
miR-370	Toll-like receptor 4 (TLR4)	U/R inhibits IL-6 and IL-1β expression and ROS levels in THP-1 cells	[111]
miR-451	14-3-3 ζ (YWHAZ)	U/R improves intimal thickening in rats following vascular injury	[112]
miR-590	Toll-like receptor 4 (TLR4)	U/R inhibited atherosclerotic lesion in ApoE-null mice and HAECS	[113]
miR-647	Phosphatase and tensin homolog (PTEN)	Upregulated in HA-VSMCs	[114]

*U/R* Up-regulation, *D/R* downregulation

Abbreviations of the cells and cell lines used in the works referenced: *HA-VSMCs* human aorta vascular smooth muscle cells, *ASMCs* human aortic smooth muscle cells, *HCASMCs* human coronary artery smooth muscle cells, *HUVECs* human umbilical vein endothelial cells, *HAECs* human aortic endothelial cells, *HMEC-1* human microvascular endothelial cell line, *ERS* endoplasmic reticulum stress

^a^All the target mRNAs have been validated by luciferase reporter assays

### Small RNAs in gene-silencing therapies

Recent years have seen a trend to develop gene-silencing, small-RNA-based, therapies to specifically target mRNAs or other miRNAs [115, 116], an approach well-suited to target undruggable targets or polygenic pathologies given the ability of small-RNAs to target multiple mRNAs and pathways [117]. The list of miRNA-based, gene silencing (or mimicking) tools is growing and includes agomirs or single-stranded miRNAs (ss-miRNAs) and antagomirs (oligonucleotides containing the complementary sequences of the target miRNA), double-stranded small-interference RNAs (ds-siRNAs), or miRNA sponges ([118] and see next section). With a growing number of possible siRNA targets in ATH research [119], several other RNA-therapies are currently in clinical trials [120]. Thus, the first siRNA-based drug (Patisiran) has recently obtained the FDA approval to silence the transthyretin (TTR) mRNA (via RNA-interference by binding its 3′UTR) which caused a rare transthyretin-mediated amyloidosis polyneuropathy originated by the deposit of TTR-protein in tissues [121]. Other miRNA-candidates for medical intervention are currently in clinical development or in phase 1 or phase 2 clinical trials, such as MRG-110, a locked nucleic acid (LNA)-modified antisense oligonucleotide against miR-92 with a potential clinical application in wound healing and heart failure [122], a miR-29b mimic (Remlarsen) to prevent formation of fibrotic scars or cutaneous fibrosis [123], or anti-miR-21 oligonucleotides, which were seen to alleviate kidney disease in a murine model of Alport nephropathy [124]. On the other hand, miRNA-mimics or antagomirs have been also used at the laboratory level to modulate miRNA expression in ATH research [125], and recently therapies directed against miR-449a [126], miR-23a-5p [109], or miRNA-98 [112], among others, have been tried in animal models with encouraging results. Lastly, therapeutic miRNAs are not restricted to targeting specific mRNAs or miRNAs, and have been also used as co-factors to limit drug resistance through silencing of key proteins promoting low drug bioavailability [127].

Nevertheless, the use of miRNAs in gene-silencing (or gene-mimicking) therapies has yet to overcome a number of difficult issues such as developing efficient delivery vehicles, reducing unwanted off-target, side effects, or blocking immune activation. Without the aim of being exhaustive (see [128–130] for recent reviews on the topic), here we will cite some of the main drawbacks associated to the design of miRNA/siRNA delivering vehicles, such as the limitation in the amount of loaded siRNA due to the rigidity of ds-siRNAs and the low surface charge of individual siRNAs that make encapsulation challenging [131]. Furthermore, conventional complexation or encapsulation with lipids nanoparticles, cationic complexes, inorganic nanoparticles, RNA nanoparticles and dendrimers introduce a significant amount of vehicle which can lead to greater potential for immunogenic response or toxicity [132]. A plausible alternative is the systemic delivery with injections or intravenous administration, since injections of miRNA drug directly into the pathogenic site have been seen to enhance target specificity, efficacy and to minimize side effects [133]. In this sense, a number of chemical modifications, e.g. with phosphorothioate, 2′-*O*-methyl-phosphorothioate, *N*,*N*′-diethyl-4-(4-nitronaphthalen-1-ylazo)-phenylamine or the LNA-nilation (locked nucleic acid) have been seen to increase stability of the DNA/RNA moiety [134]. Lastly, new strategies are being pursuit to facilitate specific delivery of the miRNA/siRNA cargo, such as the addition of targeting moieties (specific antibodies) against a protein from target cells linked to the delivery vehicle to enhance its therapeutic efficacy [135], or the “TargomiRs”, mimicking miRNAs delivered by targeted bacterial minicells [136].

On the other hand, miRNA/siRNA therapies also have the potential for silencing off-target genes, causing unexpected adverse effects due to partial sequence complementarity to 3′UTRs, this meaning a significant obstacle to the therapeutic application of miRNAs [137]. In this sense, we have recently reported that systemic treatment with an anti-CD40-siRNA increased renal NF-kB activation in the ApoE-deficient mice model of ATH (Hueso et al., J. Inflammation, in the press). Furthermore, a phase 1 trial with an anti-tumour miRNA-34 mimic (MRX34) was stopped in 2016 after severe adverse events were reported in five patients who experienced a serious immune response [116], and another phase 1 trial on patients with malignant pleural mesothelioma, treated with a TargomiRs loaded with miR-16 and targeted to EGFR, reported infusion-related inflammatory symptoms and cardiac events [138], indicating the need for more research on the impact of carriers, vehicles and therapeutic nucleic acids on the inflammatory response.

## Long non-coding RNAs (lncRNAs) and their functional relationship with miRNAs

### LncRNAs and miRNA sponges

Long non-coding RNAs (lncRNAs) represent a heterogeneous class of non-coding RNAs that includes transcripts > 200 nucleotides, which lack functional protein coding ability but modulate gene expression through multiple distinct mechanisms at epigenetic, transcriptional or post-transcriptional levels [139]. LncRNAs coordinate and integrate multiple signaling pathways and have important roles in development, differentiation, and disease [140–143]. Currently estimated at more than 56,000 [144], the number of lncRNA genes more than doubles the number of protein-coding genes in the human genome, although due their low expression levels, many lncRNAs remain poorly characterized and annotated [145], so that it is likely that this number will be increased in the years to come. Based on their presumed function lncRNAs have been classified in a number of functional groups: competitive endogenous lncRNAs (ceRNAs) and circular lncRNAs (circRNAs), with potential roles as miRNA inhibitors [146, 147], enhancer-related RNAs (eRNAs), involved in transcriptional regulation [148], transcribed ultraconserved RNAs (T-UCRs), transcribed from non-coding highly conserved genomic regions [149], and the highly heterogeneous natural antisense transcripts (NATs), intronic lncRNAS and long intergenic RNAs (lincRNAs) among others, although this classification is neither exhaustive (see [150] for a recent and comprehensive review on the topic) nor unambiguous since a lncRNA could easily fit into more than one group [151].

We are especially interested in the lncRNAs that interact with miRNAs and function as competitive inhibitors of miRNA action (“sponges”), creating loss-of-function miRNA phenotypes and causing the de-repression of its targets [152, 153]. In the next sections we will give an overview of the role of these transcripts in the regulation of miRNA function, and when data are available in ATH progression.

### LncRNAs in ATH progression and therapy: the case for ANRIL

High-throughput sequencing has allowed an exponential growth in the amount of sequence data generated in large number of individuals, and expanded the number of non-coding RNA (ncRNA) transcripts predicted to play a critical role in the pathogenesis of ATH [4] (Table 3), although because of their low expression levels, the study of lncRNAs is actually so challenging that many of them still remain poorly characterized and annotated. The lncRNA more clearly associated to ATH pathogenesis is CDKN2B-AS1, also known as ANRIL (Antisense Non-coding RNA in the INK4 locus) (see [1] for a recent review), that it is transcribed from chromosome 9p21 and acts as a lncRNA-guide to localize the polycomb repressive complex (PRC) at target promotors through a direct interaction with its subunits CBX7 or SUZ12 [154]. ANRIL is induced by the activation of the NF-kB pathway, and up-regulated ANRIL forms a functional complex with transcriptional factor Yin Yang 1 (YY1) to exert transcriptional regulation on inflammatory genes IL6 and IL8 in endothelial cells, while knockdown of ANRIL was seen to inhibit TNFα-induced expression of IL6 and IL8 expression [155], thus highlighting the involvement of ANRIL in the TNFα/NF-kB signalling that regulate inflammatory response. ANRIL expression was seen to be also correlated with a proliferative phenotype in vascular smooth muscle cells (VSMC) [156] and to act in trans, via Alu repetitive elements, to regulate other genes that participate in proatherogenic pathways [157]. Lastly, it has been reported a role for ANRIL as miRNA sponge in different tumours, such as miR-199a in triple-negative breast cancer [158], miR-186 in cervical cancer [159], or miR-323 in pediatric medulloblastoma [160].

**Table 3:** lncRNA:miRNA:mRNA axis in atherosclerosis progression

lncRNA	Sponged miRNA	Target mRNA	Regulated pathway in ATH progression	References
MALAT1	miR-204	SMAD4	Osteogenic differentiation in CAVD	[161]
MALAT1	miR-320a	FOXM1	Proliferation of HUVECs	[162]
MIAT	miR-181b	STAT3	Proliferation and apoptosis in HA-VSMC cells	[163]
MIAT	miR-149-5p	CD47	Promoted atherosclerosis progression	[164]
MEG3	miR-26a	SMAD1	Proliferation of vascular smooth muscle cells	[165]
MEG3	miR-223	NLRP3	Pyroptosis in HAEC cells	[166]
DIGIT	miR-134	Bmi-1	Viability, migration and apoptosis of HMEC-1 cells	[167]
GSA5	miR-221	MMPs	Inflammatory response in THP-1 cells	[168]
Linc00657	miR-590-3p	HIF-1α	Angiogenesis	[169]
TUG1	miR-204-5p	Runx2	Osteoblast differentiation in human aortic VICs	[170]
Linc00299	miR-490-3p	AURKA	Proliferation of vascular smooth muscle cells and HUVECs	[171]
UCA1	miR-26a	PTEN	Proliferation of vascular smooth muscle cells	[172]
Linc00305	miR-136	n.d.	Proliferation and apoptosis of HUVECs	[173]
MKI67IP-3	Let-7e	IκBβ	Inflammatory response in VECs	[174]
H19	miR-148b	WNT1	Proliferation and apoptosis of HA-VSMCs	[175]
RNCR3	miR-185-5p	KLF2	Proliferation of ECs and VSMCs	[176]

For each lncRNA shown are also a sponged miRNA and one mRNA target of this last, as well as the effect of the RNA network on ATH progression. *n.d.* not determined

Abbreviations of the tissues, cells and cell lines used in the works referenced: *CAVD* calcified aortic valve disease, *HA-VSMCs* human aorta vascular smooth muscle cells, *HUVECs* human umbilical vein endothelial cells, *HAECs* human aortic endothelial cells, *HMEC-1* human microvascular endothelial cell line, *VICs* human valve interstitial cells, *VECs* vascular endothelial cells

### Competitive endogenous lncRNAs (ceRNAs) and circular lncRNAs (circRNAs)

Competing endogenous RNAs (ceRNAs) and circular lncRNAs (circRNAs) could be described as the “professional” miRNA “inhibitors/sponges”, i.e. the families of lncRNAs that work as “dominant negatives” of miRNA action by interacting with their seed regions to potentially block whole families of related miRNAs [152, 177]. This interaction cause the de-repression of their downstream targets, because miRNA-target interaction is strongly concentration-dependent [178], and has been seen to be de-regulated in a number of pathological conditions, from cancer to neurodegenerative diseases [179, 180]. CircRNAs are generated, by the thousands, from exonic or intronic regions in mammalian cells by a back-splicing event that links covalently the 3′ and 5′ ends of the transcript, so that they do not have a 5′ cap or 3′ tail [181, 182], and their expression is submitted to tissue/developmental-stage-regulation [183]. In recent years a number of groups have reported on the impact of lncRNA-sponges on ATH and related cardiovascular conditions with a mechanistic detail that include co-regulated miRNA and mRNA targets [184–186], and this is becoming a hot topic in cardiovascular research (see Table 3).

### Transcribed ultraconserved RNAs (T-UCRs)

The first T-UCR RNA to be described was Evf-2, transcribed from the ultraconserved region between the homedomain containing genes Dlx-5 and Dlx-6. At the functional level, Efv-2 works as a coactivator of Dlx-2 to increase the activity of the transcriptional enhancer close to the Dlx-5/6 cluster [187]. Expression of T-URCs is tightly regulated, and a number of them (Uc.160+, Uc283+A and Uc.346+, Uc for Ultra Conserved) were seen to be silenced through DNA methylation at specific CpG islands in transformed cells [188]. Other disease associated T-UCRs have been detected, mainly in tumours, thus Uc.416+A was seen to be upregulated in renal cell carcinoma [189], as Uc.383 in hepatocellular carcinoma [190], Uc.338 in colorectal cancer [191], or Uc.63 in breast cancer ([192], see also [149] for a recent review). Interestingly, a number of authors have reported regulatory interactions among T-UCRs and miRNAs. In this sense, it was seminal the report of a direct interaction of Uc.283+A with pri-miR-195 that prevented the cleavage of this last by Drosha and hindered its maturation [193]. Subsequently, other authors have described further T-UCR/miRNA interactions such as that of Uc.173 with miRNA-195 [194] or miR-29b [195] to facilitate function of the intestinal epithelium, or the interaction of Uc.416+A with miR-153 in renal cell carcinoma [189].

### Natural antisense transcripts (NATs)

NATs is a highly heterogeneous group of lncRNAs, transcribed from the complementary chain of target genes in an antisense orientation, that regulate post-transcriptionally gene expression via RNA:RNA interactions with mRNA or miRNAs [196]. In this sense, oncogenic lncRNA FOXD1-AS1 (FOXD1-antisense 1), the antisense transcript of the gene FOXD1, was reported to interact with miR339-5p and miR342-3p [197], tumor suppressor TP73-AS1 sponged miR-941 [198], while TSPAN31, the natural antisense transcript of cyclin dependent kinase 4 (CDK4), interacted with miR-135b in hepatocellular carcinoma causing TSPAN31 silencing and the subsequent upregulation of CDK4 [199].

### Retrogressed genomic elements: processed pseudogenes and Alu repeated elements

Retrogressed genomic elements conform an heterogeneous group of expressed mRNAs that have made their way back into the genome through retrogression, i.e. a cycle of retrotranscription (mRNA to cDNA), and insertion (cDNA into genomic DNA) catalysed by the reverse transcriptase and endonuclease activities of the LINE retrotransposons [200]. Among them the best characterized are the processed pseudogenes, originated by the retrogression of a functional mRNA, and the repeated sequences of the Alu family, a member of the Short Interspersed Nuclear Elements (SINEs) group that come from a founder Alu element.

Processed pseudogenes underwent 3′-end polyadenylation and do not contain introns, since they come from fully-spliced transcripts, are flanked by duplicated integration sites 5 to 20 bp in length and upon genomic integration they suffer a process of sequence degeneration [201]. Pseudogenes were initially considered as the paradigm for “junk DNA” since these were genes (mRNAs) that lost its coding function, but recent works have re-evaluated their function and now it is widely accepted that they have a role in the regulation of gene expression and that its dysregulation is often associated with various human diseases including cancer [202]. According to last estimates, the number of processed pseudogenes in the human genome is similar to that of “true” coding genes [201], and some of them have been seen to function as miRNA sponges [203]. Although expressed pseudogenes could be considered as the perfect miRNA sponges since they provide mostly homologous miRNA binding sites in the correct sequence context, leading to the paradox that expression of the pseudogene could regulate expression of its corresponding gene [204], there are several constraints that could impact on the role of pseudogenes in miRNA function. Thus, the sequence degeneration subsequent to the integration of pseudogenes in the genome might inactivate miRNA binding sites, while the genomic context of the integration site could impose patterns of expression different from those of the parental gene. Nevertheless, the most critical factor is the difference in gene-number among the parental gene and its pseudogene progeny since not all genes have their corresponding expressed pseudogenes while a number of them are overrepresented in the pseudogene count, as the 2090 pseudogenes found for the 79 genes encoding human ribosomal proteins, from which 145 pseudogenes correspond to the RPL21 [205]. Despite these constraints, several groups have characterized different pseudogenes as miRNA sponges, and a manually curated database (miRsponge) has been created [203]. Thus PMS1 Homolog 2, Mismatch Repair System Component Pseudogene 2 (PMS2L2) has been described as a molecular sponge of miR-203 in osteoarthritis, with MCL-1 mRNA being the direct target of miR-203 [206], ferritin heavy chain 1 pseudogene 3 (FTH1P3) was shown to suppress miR-206 activity to promote ABCB1 (ATP binding cassette subfamily B member 1) protein expression [207], and to sponge miR-224-5p to modulate expression of fizzled 5 [208]. Furthermore, OCT4-pseudogene 4 was shown to protect OCT4 mRNA from miR-145 [209], and PTENp1 (PTEN pseudogene 1) was seen to shield PTEN mRNAs from miR-21 in oral squamous cell carcinoma (OSCC) [210], and from miR-106b and miR-93 in gastric tumours [211].

**Fig. 3:**
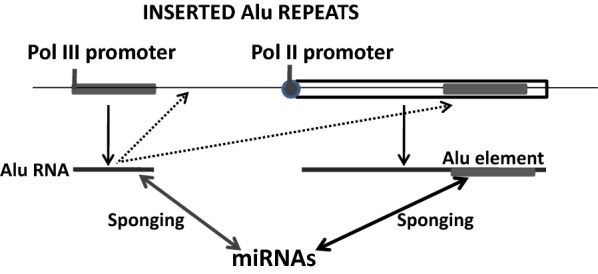
Role of Alu elements in the regulation of miRNA activity. Shown are nuclear Alu elements transcribed from its own RNA pol III promoter in the case of being independent transcriptional units (left), or from an RNA pol II promoter in the case of being integrated inside another gene. In both cases these can behave as miRNA sponges by interacting with miRNAs. Some individual Alu elements can retroinsert into intergenic regions or inside other transcriptional units (taken from [1])

On the other hand, the other group of RNA dark transcripts that are also retrogressed to the genome and function as miRNA sponges is that of Short Interspersed Nuclear Elements (SINEs) [212]. SINEs include the Alu repeated sequences, a family of highly successful genomic parasites that have colonised the human genome to the extent that over 10% of it (i.e. one million copies) is composed by Alu-derived sequences (see [1] for a recent review). Alu repeats incorporated to the human genome from a founder element by using the reverse transcriptase encoded in LINEs [213, 214], and have subsequently undergone a process of sequence degeneration that has inactivated their transpositional ability, leaving only a few active members in the genome [215]. Genomic Alu elements include a RNA polymerase III internal promoter at the 5′ end of left arm and a short poly-A tail at the 3′ end of the right arm [216]. Although most of the members of the Alu family are silenced in the human genome, some of them are transcribed by RNA polymerase III into free Alu RNAs, as concatemers of individual Alu-RNAs by a yet unknown mechanism, or by RNA pol-II as mRNA-embedded Alus [216, 217], this last being a significant source of expressed Alu elements since aprox. 30% of human genes harbour a copy of an Alu repeat, usually at their 5′ or 3′ UTRs [218] (Fig. 3).

Although the functional relationships among Alu elements and miRNAs are complex and poorly understood, mostly due to the dual nature of Alu repeats as free transcripts or mRNA-inserted sequences, it is evident that the presence of the highly homologous, Alu repetitive sequences in different mRNAs could supply a platform of common binding sites for their coordinated targeting by miRNAs or to act as miRNA sponges [219]. In this sense, it has been reported a subset of 3′UTRs which included Alu elements with strong potential target sites for over 50 different miRNAs [220], and a group of 30 miRNAs that showed short-seed homology with highly conserved Alu elements at the 3′ UTRs of human mRNAs [221]. Furthermore, miR-15a-3p and miR-302d-3p, were recently shown to target RAD1, GTSE1, NR2C1, FKBP9 and UBE2I exclusively within Alu elements [222], while miR-661 caused the downregulation of Mdm2 and Mdm4 by interacting with Alu elements in their sequence [223], and Di Ruocco et al. described an Alu RNA that functioned as a miR-566 sponge [224].

## Unveiling RNA: RNA regulatory networks in the progression of atherosclerosis

Establishing RNA:RNA regulatory networks that included mRNAs and miRNAs (and lncRNAs) would facilitate our ability to use them for research and therapeutic purposes. In this context, we could consider the miRNAome as a “safety net” to preserve homeostatic levels of mRNA expression, while lncRNA sponges would contribute to maintain regulatory levels of miRNAs. In any case, mRNAs, miRNAs and sponging lncRNAs conform RNA:RNA regulatory networks that are based on their direct physical interaction, which in turn depends on the sequence homology.

The first requisite for constructing a regulatory network is to identify the mRNA targets of a specific miRNA (or miRNA signature), and the most direct way to study these direct interactions is by isolating hybrid duplexes. A number of methods have been designed for this purpose, most of them variations of a basic miRNA/target cross-linking and immunoprecipitation (CLIP) assay followed by sequencing, such as HITS-CLIP [225], miR-CLIP [226], AGO-RIP-Seq [227], LIGR-Seq [228], Biotin-Pulldown and RNA-seq [229] etc. (see [230] for a recent review on the topic). Once miRNA/mRNA pairs have been identified with any of the above methods, the interaction is then confirmed by a luciferase assay in which the 3′UTR of the mRNA to test is cloned downstream a luciferase gene and the silencing ability of the miRNA is measured as changes in the light emitted by the construct [231]. Nevertheless, all these methods are complex, cumbersome and time-consuming, and are poorly suited for the clinical laboratory, so most of the miRNA work is currently performed by using bioinformatic algorithms that define miRNA/mRNA interactions (and predicts miRNA targets) after measuring sequential, structural or thermodynamic features (see [232] for a comprehensive review on the topic). Recent years have seen the proliferation of algorithms and web servers designed to predict miRNA targets [233], of which a few have established themselves as reference tools ([234] and see Table 4). Although it is not the aim of this review to make a comparative analysis of these algorithms (see [235] for a recent review on this topic), it is widely accepted that their predictions are frequently inconsistent, inaccurate and plenty of false positives [236, 237]. One answer to this problem has been the development of secondary algorithms that perform a more comprehensive analysis by combining the outputs of a number of primary target predictions (e.g. miRSystem combines seven primary algorithms while miRWalk2.0 combines 12 of them) and allow to control the stringency of the search by setting the number of common hits required for a prediction to be considered as positive [238, 239], but the output of these analysis also are long lists of predicted targets. The answer to overcome these problems has been the development of integrated/enrichment analysis which are well suited to deal with long lists of genes, though the results obtained are not so solid as those from experimental assays. In the integrated analysis, the list of predicted targets is pruned by establishing additional conditions to confirm targets [240, 241]. Although there are different approaches depending on the kind of sequence information available, a typical experiment would compare the entire list of predicted targets for a single miRNA or a miRNA signature with a list of Differentially Expressed Genes (DEGs) from the same experimental background or from an expression repository such as the Gene Expression Omnibus (GEO) [242], and the entries common to both lists would be saved. In a second step, those showing a counter-regulated expression vs. the miRNA/s (i.e. inversely correlated expression levels) would integrate a restricted list of preferential putative targets (Table 4 shows different algorithms for integrative analysis). By using a similar approach, Zhang et al. have recently constructed a miRNA:mRNA regulatory network for ATH progression in icariin-treated, high-fat fed ApoE-deficient mice which showed that changes in miRNA expression mainly affected the PI3K/Akt, Ras, ErbB and VEGF signalling pathways in lesions [243].

**Table 4:** Resources for miRNA research

Algorithm/database	Web address	Utility	References
miRBase	www.mirbase.org	MiRNA database	[250]
miRTarbase	mirtarbase.mbc.nctu.edu.tw/php/index.php	MiRNA target interaction database	[251]
Tarbase	www.microrna.gr/tarbase (& follow link)	MiRNA target interaction database	[252]
DIANA	diana.imis.athena-iinnovation.gr/DianaTools/	MiRNA research tools	[253]
doRiNA	https://dorina.mdc-berlin.de	Database of RNA interactions	[254]
miRanda	https://omictools.com/miranda-tool	MiRNA target prediction. No longer	[255]
mirBridge	n.a.	MiRNA target prediction	[256]
miRDB	www.mirbd.org	MiRNA target prediction	[257]
miRmap	https://mirmap.ezlab.org	MiRNA target prediction	[258]
miRNAMap	mirnamap.mbc.nctu.edu.tw	MiRNA research tools	[259]
PicTar	https://pictar.mdc-berlin.de	MiRNA target prediction	[260]
PITA	http://genie.weizmann.ac.il/pubs/mir07/mir07_prediction.html	MiRNA target prediction	[261]
RegRNA	regrna2.mbc.nctu.edu.tw/	RNA-RNA interaction regions	[262]
rna22	https://cm.jefferson.edu/rna22/	MiRNA target prediction	[263]
RNAhybrid	https://bio.tools/rnahybrid	MiRNA target prediction	[264]
Targetscan	www.targetscan.org	MiRNA target prediction	[77]
miRSystem	mirsystem.cgm.ntu.edu.tw/	Comprehensive analysis	[238]
miRWalk2.0	zmf.umm.uni-heidelberg.de/apps/zmf/mirwalk2/	Comprehensive analysis	[239]
CORNA	omictools.com/cornas-tool (& follow link)	Integrated analysis	[265]
MMIA	epigenomics.snu.ac.kr/MMIA/public_html/index.html	Integrated analysis	[266]
miARma-seq	http://miarmaseq.idoproteins.com/	Integrated analysis	[241]
DIANA-LncBase	carolina.imis.athena-innovation.gr/diana_tools/web/index.php?r=lncbasev2%2Findex	Predicted microRNA targets on lncRNAs	[267]

Shown are algorithms and databases for the prediction of miRNA targets, and for the comprehensive and integrated analysis of miRNA/mRNA interactions (see main text). This is not an exhaustive list, and the selection only reflects author’s preferences

The process of delineating RNA regulatory networks has been further facilitated by the development of the Gene Ontology (GO) enrichment analysis in which individual genes from a set of Differentially Expressed Genes (DEGs) from a stated condition are classified in pre-defined categories (GO-terms) to identify those that account for more DEGs (enrichment) [244]. These will define gene networks as structural pathways or molecular functions specific for that condition. GO analysis on ATH-DEGs has showed an enrichment in proteins related to nucleic acid function, such as epigenetic regulators [245], [liver X] nuclear receptors [246], or ribosomal proteins [247], while our own GO analysis on a subset of miRNA targets obtained after an integrated analysis in ATH also showed an enrichment in genes related to the function of nucleic acids (Hueso et al., manuscript in preparation).

Lastly, inclusion of lncRNAs in the efforts to delineate disease-related regulatory networks greatly increases their complexity, not only because this means the inclusion of new players in the game but also because lncRNAs are very heterogeneous in function and can act at different levels as miRNA sponges, compete with miRNAs for shared mRNA targets, or interact with the chromatin structure, facts that greatly hinder their functional characterization. Furthermore, information on the function of individual lncRNAs is scant and incomplete for most of them, since only a few hundreds of lncRNAs have been yet functionally characterized, and for most of them the detailed mechanisms of action are still to be determined. Nevertheless, a number of groups have reported mutual miRNA:mRNA:lncRNA interactions in the context of ATHp ([248, 249] and see Table 3).

## The dark transcriptome in clinics: future challenges

One of the most striking consequences of the completion of the human genome has been the conversion of the dark transcriptome (encoded by the “junk” DNA) into an elaborated catalogue of regulatory RNAs, many of them related to the onset and progression of human diseases. In this sense, the next challenge is to make profit of this ncRNA revolution in the clinical context to explore their role as specific biomarkers or as etiopathogenic intermediates, but this will require new technical developments on the way that sequencing information is generated, managed and interpreted.

For many years, the mantra of the sequencing industry has been “faster, longer and cheaper”, and it is likely that this will be also the aim for the development of the next generation of sequencing machines with the addendum of giving extra importance to accuracy. Sequencing ncRNAs up to clinical analytical standards is not an easy business since it requires an unprecedented degree of accuracy and flexibility. Accuracy because detecting point mutations in ncRNAs (critical for cancer research) cannot be compromised by the technical noise from the reagents used for amplifying and generating the sequence or from the machine used to detect it [268], and performing multiplex sequencing in a sample is not the solution since this significantly increases the costs associated to the process. Flexibility, because ncRNAs are very heterogeneous in size and structure, with many events of alternative splicing that originate multiple, partially homologous, forms that suppose a challenge to reconstruct long sequences from short reads. Sequencing genomic regions rich in clustered repetitive sequences (e.g. Alu repeats) pose a similar problem that can only be solved by increasing the length of the sequence reads without compromising accuracy. Nevertheless, the sequencing industry has demonstrated to be innovative and dynamic, and although at this time it is difficult to ascertain which of the sequencing platforms currently in use will rule in the next future, whether different platforms will specialize in specific niches, or if there’s yet to come a new and disruptive technology, we can give for sure that this problem is being addressed.

The second big challenge to introduce ncRNA expression profiling in the clinical context has to be with the way that the sequencing information generated is managed and used. On the one hand, all this information has to be stored in a way that can be easily retrieved, and new software has to be developed to extract biological or medical “sense” from it. Furthermore, the problem of data compatibility and standardization is always behind the door. With many different sequencing platforms in the market (and other many to come in the future) developers should make an effort to share standards and avoid proprietary data formats, to encourage data sharing and to provide public, non-commercial and unrestricted access to data. Failure on doing this will lead us to a nightmare of data islands. On the other hand, data interpretation at the whole genome/transcriptome level will surely require using artificial intelligence and deep learning algorithms for the analysis and to discover new biological insights from sequencing data. Genomic datasets are too large and complex to be mined by individual researchers looking for pairwise correlations, so that the need for new and potent analytical tools is clear. Machine learning and deep learning, a subdiscipline of machine learning, are powerful tools suited to data-driven sciences that are currently used to automatically explore the genome and detect patterns in data that could be used to unravel novel properties of noncoding regions and to understand how they impact in human health [269, 270]. The strong flexibility and high accuracy of deep learning methods is supported by the successive introduction of a variety of deep architectures that are superior over other existing methods. In this sense, Splice AI, a deep neural network, has been used to predict splice junctions from a pre-mRNA transcript, as well as noncoding variants with the ability to cause cryptic splicing events [271]. It is likely that many other similar algorithms will be developed to assist the analysis of whole transcriptomes/genomes.

## Conclusions

We are on the verge of a new revolution in the way we see disease and the normal, non-diseased state. For many years, diseases have been linked to mutations in the genomic DNA or to alterations in the expression of coding mRNAs. We now know that this “coding world” is just the tip of the gene expression iceberg. It is not only that there are more non-coding RNAs than coding ones, but that all these RNAs interact among them (and with chromatin), to create complex regulatory miRNA/lncRNA/mRNA networks whose unbalance underlies the basis of complex diseases. Constructing accurate models of disease, a requisite for developing new and personalized treatments, will require new developments to generate accurate sequencing information as well as to make this information manageable and available to all ranks involved in alleviating the burden associated to human diseases.

## Data Availability

Not applicable.

## Abbreviations

ATH: atherosclerosis
ceRNAs: competing endogenous RNAs
circRNAs: circular RNAs
DEGs: Differentially Expressed Genes
EST: expressed sequence tag
LINEs: long interspersed nuclear element
lncRNAs: long non-coding RNAs
miRNAs: microRNAs
ncRNAs: non-coding RNAs
NGS: next generation sequencing
SINEs: short interspersed nuclear element
3′UTR: 3′ untranslated region
